# Holographic free-electron light source

**DOI:** 10.1038/ncomms13705

**Published:** 2016-12-02

**Authors:** Guanhai Li, Brendan P. Clarke, Jin-Kyu So, Kevin F. MacDonald, Nikolay I. Zheludev

**Affiliations:** 1 Optoelectronics Research Centre & Centre for Photonic Metamaterials, University of Southampton, Southampton SO17 1BJ, UK; 2 National Key Laboratory for Infrared Physics, Shanghai Institute of Technical Physics, Chinese Academy of Sciences, 200083, China; 3 Centre for Disruptive Photonic Technologies, School of Physical and Mathematical Sciences & The Photonics Institute, Nanyanag Technological University, Singapore 637371, Singapore

## Abstract

Recent advances in the physics and technology of light generation via free-electron proximity and impact interactions with nanostructures (gratings, photonic crystals, nano-undulators, metamaterials and antenna arrays) have enabled the development of nanoscale-resolution techniques for such applications as mapping plasmons, studying nanoparticle structural transformations and characterizing luminescent materials (including time-resolved measurements). Here, we introduce a universal approach allowing generation of light with prescribed wavelength, direction, divergence and topological charge via point-excitation of holographic plasmonic metasurfaces. It is illustrated using medium-energy free-electron injection to generate highly-directional visible to near-infrared light beams, at selected wavelengths in prescribed azimuthal and polar directions, with brightness two orders of magnitude higher than that from an unstructured surface, and vortex beams with topological charge up to ten. Such emitters, with micron-scale dimensions and the freedom to fully control radiation parameters, offer novel applications in nano-spectroscopy, nano-chemistry and sensing.

## Abstract (web summary)

Controlling the generation of light in nano-scale systems is a challenging task and is of growing importance. Here, Li *et al*. propose a means of controlling the wavefront of light emanating from a single nano scale emitter by holographic principles using a plasmonic metasurface.

## Graphical abstract

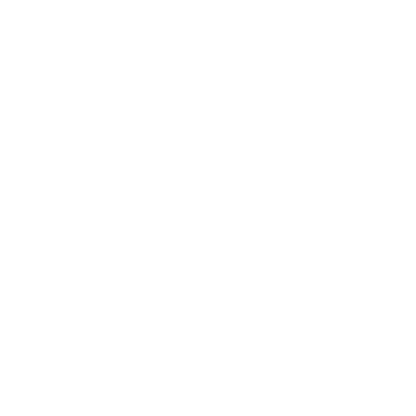

A diverse toolkit of meso-/macroscopic optical elements exists to control and manipulate light, that is its amplitude, polarization, photon energy and momentum, in free-space and photonic waveguide systems. Recent advances in nanofabrication technologies and improved understanding of near-field light/matter/free-electron interactions are now enabling the extension of such control to the nanoscale: the coupling of light into well-defined free-space modes of photon energy, momentum and polarization has been made possible with the help of photonic crystals^1^, surface waves^2^,^3^,^4^, nanoantennas^5^,^6^,^7^ and photonic metamaterials^8^,^9^. Specifically in relation to electron beam excitations, plasmonic nanoantennas^10^,^11^,^12^, cylindrical metal-dielectric ‘undulators'^13^ and collective oscillating metamolecule ensembles^14^ have been engaged to such ends. Regardless of the mode of excitation, structures are typically constrained to controlling the direction and polarization of emitted light.

Holography was originally conceived as a technique for increasing the resolution of scanning electron microscopes^15^, but has come to be widely recognized as the ultimate method of achieving three-dimensional optical reconstruction of objects. In essence, it relies on the encoding of an ‘object' wavefront in a recorded interference pattern produced with a ‘reference' wave; the object wave can then be reconstructed by illuminating the interference pattern with the reference wave. Its conceptual simplicity sees the technique employed routinely for wavefront conversion of light^16^,^17^ and matter waves^18^,^19^, where the required interferogram is computationally generated.

In this work, we propose a flexible means of precisely controlling the wavefront of light emanating from a singular nanoscale emitter by locating it in a nanostructured environment designed according to holographic principles (Fig. 1). Surface nanostructures are engineered to convert the divergent transition radiation and surface plasmon polaritons emanating from the impact point of an electron beam on a metal surface into light beams with selected wavefronts, specifically directional plane-waves and high-order optical vortex beams.

## Results

### Holographic design for electron-induced emission control

**Figure 1:**
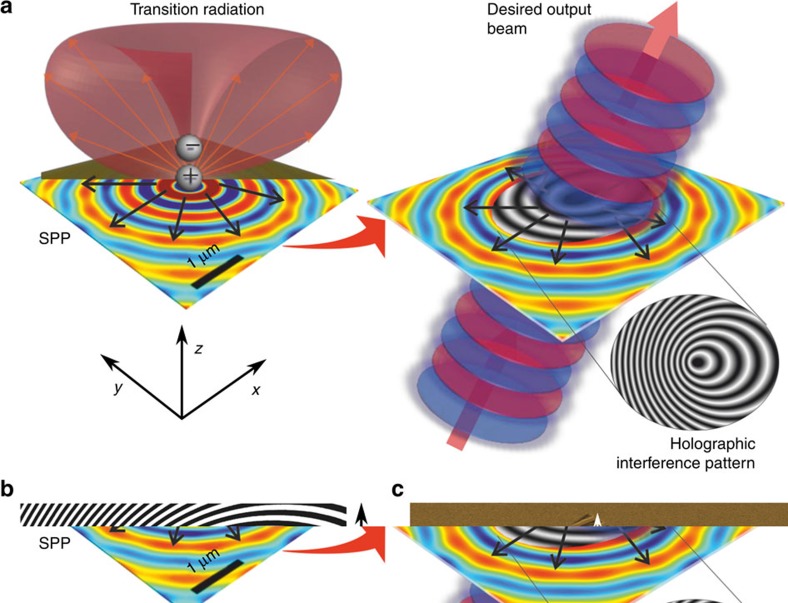
Holographic free-electron light source design. (**a**) The electromagnetic excitation resulting from normally incident free-electron impact on a metal surface is computationally replicated by an electric dipole located in close proximity to the surface. The holographic mask required to couple this excitation to a desired output beam is obtained via the interference of the dipole-generated near-field and the required output field, as schematically illustrated for a collimated plane wave at an oblique angle to the surface-normal. (**b**) Binary version of the as-generated greyscale interference pattern required to produce, from the impact of 30 keV electrons on a gold surface, an output beam at a wavelength of 800 nm directed at 30° to the surface-normal. (**c**) False colour scanning electron microscope image of the pattern from panel (**b**) fabricated on an optically thick (140 nm) gold film (scale bar, 10 μm).

A charged particle crossing the boundary between two different media generates transition radiation (TR)^20^, with a spectral distribution and intensity related to the relative permittivities of the media and the electron energy. On metal surfaces such impacts also generate surface plasmon polaritons (SPPs) propagating radially from the impact point. Indeed, for certain metals at certain frequencies and electron energies the efficiency of coupling to SPPs may be greater than to TR, but SPPs can only contribute to free-space (far-field) light emission in the presence of a decoupling structure such as a grating; TR is otherwise the dominant output component^21^,^22^. The TR from an electron normally incident on a metal surface has a cylindrically symmetric toroidal emission pattern (illustrated schematically in Fig. 1a) very similar to that of a dipole aligned with the surface-normal (at a distance from the surface *h<<λ*, where *λ* is the light wavelength). Indeed, for numerical modelling purposes, an electron impact excitation on a metal, including SPP generation, can be accurately represented as such^21^,^22^,^23^,^24^.

We select an emission design wavelength *λ*=800 nm for the present study, which in experiment utilizes 30 keV electrons normally incident on gold surfaces. The corresponding numerically simulated distribution of electromagnetic field in the gold/vacuum interface plane is employed as the ‘reference' field (in holographic parlance) to generate an interference pattern with an ‘object' light field corresponding to the desired output wavefront, which by inversion defines the 2D surface structure required to regenerate the object (output) beam from an electron-impact excitation. For example, a plane-wave object beam propagating at a polar angle of 30° to the surface-normal produces the pattern of offset concentric oval rings shown inset to Fig. 1a. The interference pattern obtained is converted to a binary mask^25^,^26^ (Fig. 1b) for ease of fabrication by focused ion beam milling on an optically thick (140 nm) polycrystalline gold film (Fig. 1c) deposited by resistive evaporation. It should be noted that the computed holographic (interference) patterns are two-dimensional, that is they provide no information on the required height/depth of surface-relief features for optimal coupling efficiency, which will generally depend on the relative efficiencies of SPP and TR generation^21^, emission wavelength and polar angle^27^. In the present case, features are etched to a depth of 60 nm, which is found, both computationally (in simulations of a binary gold surface-relief hologram driven by a dipole source at *h*=50 nm, constrained to a 10 μm × 10 μm *xy* domain) and experimentally, to maximize 800 nm emission intensity at *θ*=30°.

### Directional plane-wave emission

**Figure 2:**
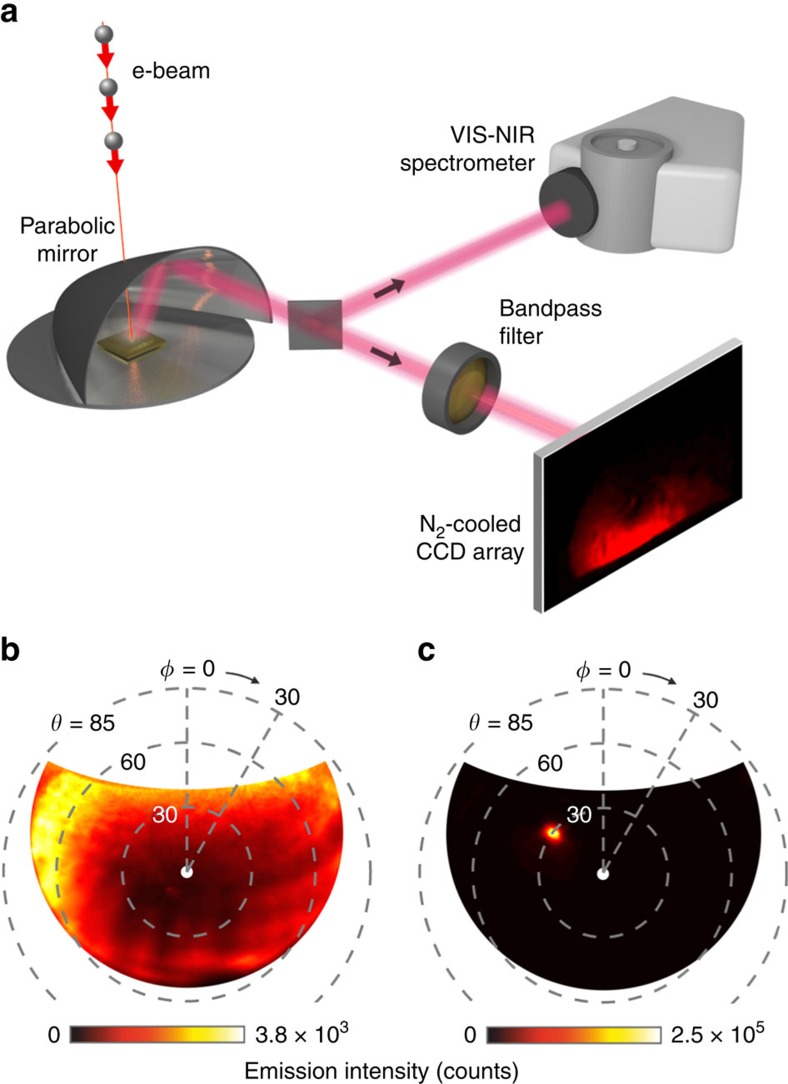
Angle-resolved spectroscopy of electron-induced light emission. (**a**) Schematic of the scanning electron microscope-based system for angle-resolved electron-induced light emission spectroscopy. Electrons impinge on samples through a small hole in a parabolic mirror, which collects and collimates emitted light, the beam being subsequently directed to either a spectrometer or imaging CCD (for simplicity, lenses/mirrors/apertures in these paths are not shown). (**b**,**c**) Angular distribution of 800±20 nm light emission induced by electron-beam impact (**b**) on an unstructured gold surface and (**c**) at the centre of a 30 μm × 30 μm holographic mask in gold, designed to produce a plane wave 800 nm output beam at *θ*=30° (the azimuthal angle *ϕ* being arbitrarily set by in-plane sample rotation beneath the incident electron beam; signals are integrated over a 20 s sampling period).

Electron-induced radiation emission spectroscopy and imaging have emerged in recent years as powerful tools, variously with nanometre spatial and picosecond temporal resolution, for the characterization of plasmonic modes, crystallographic defects, carrier dynamics, luminescence lifetimes and the local density of optical states in photonic and optoelectronic nanostructures^14^,^21^,^22^,^28^,^29^,^30^,^31^,^32^,^33^. In the present case, the spectral and directional distribution of electron-induced light emission from holographic structures is characterized in a scanning electron microscope operating in fixed-spot mode, with an electron energy of 30 keV and beam diameter of 50 nm. Emitted light is collected by a parabolic mirror located above the sample and directed to either a VIS/NIR spectrometer or a CCD camera configured to image the parabolic mirror surface, that is to map the angular distribution of light emission (Fig. 2a).

Figures 2b,c present the 800±20 nm light emission distributions for electron injection, at a beam current of 12 nA, respectively on the unstructured gold surface and at the centre of the holographic nanostructure shown in Fig. 1c. (The asymmetry seen in the broadly divergent transition radiation pattern from the unstructured surface—Fig. 2b—is an instrumental artefact related to mirror alignment imperfection and does not depend on sample rotation or polycrystalline domain orientation.) In stark contrast to the flat gold surface, the holographic nanostructure produces strongly directional emission at *θ*=30°, as per design (Fig. 2c) with a peak emission intensity in the selected direction that is increased by around two orders of magnitude.

**Figure 3:**
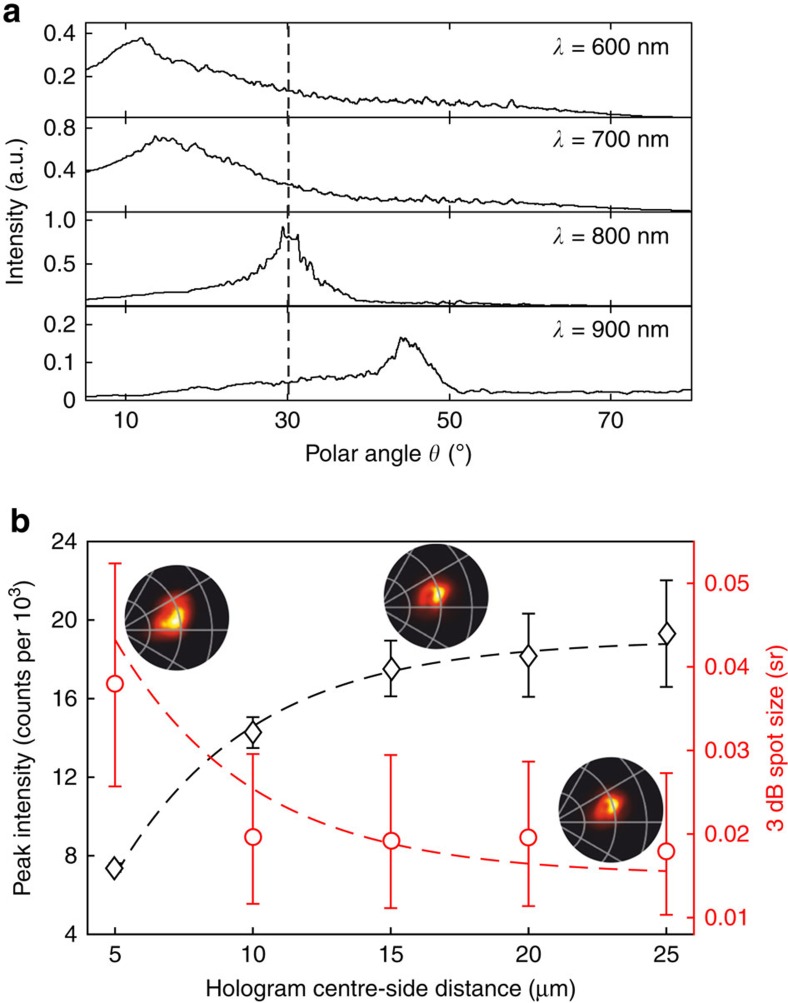
Performance of gold surface holographic light sources. (**a**) Emission intensity as a function of polar angle *θ* (integrated over all azimuthal angles) in 40 nm wavelength bands centred at 600, 700, 800 and 900 nm, for a 30 μm × 30 μm pattern engineered for 800 nm emission at *θ*=30° (electron beam current=6.1–6.3 nA; integration time=30 s). (**b**) Peak intensity and 3 dB full-width half-maximum spot size of 800±20 nm light emission (evaluated at the brightest pixel and as solid angle around said pixel respectively) as functions of the in-plane dimensions of the holographic structure in microns (beam current=5.0–5.2 nA; integration time=30 s; dashed trend lines are exponential curves with a growth/decay constant of 20 μm; Error bars for intensity are evaluated as the difference between the peak pixel intensity and the peak of a Gaussian fit to the polar cross-section through that pixel; and for spot size by including ±1 pixel either side of the 3 dB boundary, weighted for relative brightness, in the evaluation of solid angle). Sections of angular emission intensity distributions are shown inset for holograms with centre-edge distances of 5, 15 and 25 μm (polar and azimuthal grids are in 15° and 30° steps respectively).

The holographic nanostructure is essentially a diffractive element, which implies that it should exhibit a dispersive response for light emission at wavelengths other than the design wavelength. This is illustrated in Fig. 3a, where emission intensity integrated over all azimuthal angles is plotted as a function of polar angle for emission wavelengths, 600, 700, 800 and 900±20 nm. Shorter/longer wavelengths are directed at smaller/larger polar angles respectively, with peak emission intensity falling on either side of the structural design wavelength as the phase mismatch among waves scattered at different locations on the structure grows. One would also expect the performance of holographic nanostructures to depend on their size, that is on the number of constituent scattering elements, and this is clearly seen to be the case: Fig. 3b shows peak 800 nm emission intensity and spot size as functions of in-plane pattern size for a set of hologram dimensions from 10 μm × 10 μm to 50 μm × 50 μm. With increasing pattern size, Emission intensity and spot size (c.f. beam divergence) increase and decrease respectively at a rate consistent with the ∼25 μm exponential propagation length of SPPs on polycrystalline gold surfaces at the design wavelength^34^,^35^.

### Vortex beam generation

**Figure 4:**
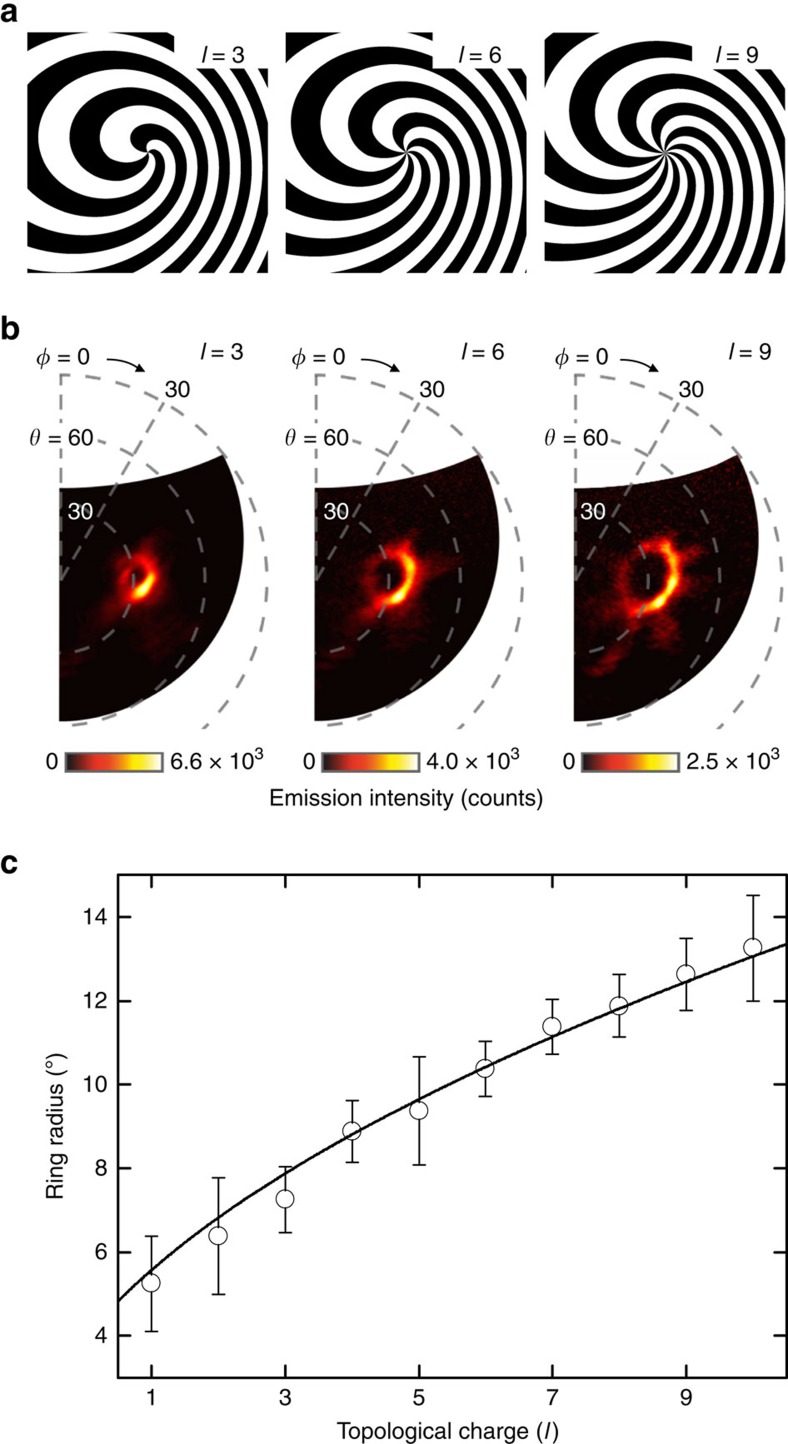
Generation of optical vortex beams. (**a**) Central portions of binary holographic masks for electron-beam induced generation of optical vortex beams with topological charge *l*=3, 6 and 9 and (**b**) corresponding angle-resolved emission intensity distribution maps (electron beam current=5.5–6.1 nA; integration time=30 s). (**c**) Mean radius of the ring-shaped vortex beam intensity profile (averaged over polar and azimuthal directions with s.d. error bars) as a function of topological charge *l*, with a fitting of the form *A(l+1)*^*1/2*^. The factor *A* is employed here as a fitting parameter, taking a value 3.937° (analytically^40^ it should depend on wavelength and propagation distance from the vortex beam source).

The holographic design approach can readily be applied to generate more complex wavefronts than the plane-wave considered thus far. To illustrate this, we fabricated holographic nanostructures encoded with optical vortex beams^36^. Such beams have a phase that varies in a corkscrew-like manner along the direction of propagation, described by azimuthal phase dependence e^*ilϕ*^, where *ϕ* is the azimuthal angle with respect to the beam axis and *l* is an integer known as the topological charge (*l*=0 representing a plane wavefront). They are non-diffracting, have a characteristic ring-shaped intensity profile and carry orbital angular momentum that can be transferred to illuminated objects, making them particularly interesting for optical trap and tweezers applications^37^,^38^. Holographic structures were designed to generate optical vortex beams of varying topological charge, again at a polar angle *θ*=30° and a wavelength of 800 nm, for an electron energy of 30 keV. These comprise patterns of interlocking spiral arms, with the number of arms corresponding to the topological charge, as illustrated in Fig. 4a for *l*=3, 6 and 9. Fig. 4b shows the far-field distribution of the light emitted from holographic nanostructures of *l*=3, 6 and 9 respectively. The observed ring-shaped intensity profile, with a central intensity null arising as a consequence of the phase singularity on the beam axis, is a defining characteristic of vortex beams. The expected increase in ring radius with topological charge, as *∼[l+1]*^*1/2*^ (analytically derived in ref. 39), is very clearly seen in Fig. 4c.

## Discussion

In summary, we have shown experimentally that holographically nanostructured surfaces can be employed to control the wavefront of light emission from nanoscale sources. The concept is demonstrated in application to the transition radiation and surface plasmon polaritons generated by electron beam impact on a metal (gold) surface, with holographic patterns engineered to produce directed, low-divergence, directional plane wave and optical vortex beams. Brightness (evaluated in photons per unit solid angle per electron) at the design wavelength is enhanced by as much as two orders of magnitude in the present case, and stronger enhancement may be achieved at shorter wavelengths where electron-induced excitation of SPPs is more efficient.

The control of energy transfer and conversion, in particular the generation of light, in nanoscale systems is a technological challenge of great and growing importance. With micron-scale dimensions and the freedom to fully control radiation parameters, holographic free-electron light sources offer novel applications in such areas as nano-spectroscopy, nano-chemistry and sensing. With an appropriate reference field model, the approach can be adapted to a variety of nanoscale ‘point source' emitters, for example quantum dots and fluorescent molecules, to other substrate media, and to higher electron energy domains. The use of continuously-variable-depth, as opposed to binary, holographic nanostructures and phase-gradient metasurface patterns^40^ may add additional degrees of freedom to the control of emission spectrum/wavefront and device efficiency without the complications of multilayer device architectures.

## Methods

### Numerical simulation of electron-induced fields

For the purposes of designing holographic surface structures, the distribution of electromagnetic field in the gold/vacuum interface plane resulting from free-electron injection are computed using the model of a fixed dipole above the surface^21^,^22^,^23^,^24^, via the finite element method (in COMSOL Multiphysics). We select a surface–dipole separation *h*=50 nm (*λ*/16) that is small enough to correctly reproduce the electron-induced fields (there is negligible variation in the transition radiation distribution among values of *h*<*λ*/8, that is <100 nm in the present case) whilst being larger than the local finite element mesh size (20 nm), so as to avoid anomalies in simulated electromagnetic field distributions. The gold and vacuum are assumed to be semi-infinite in the *z*-direction, with material parameters for gold taken from ref. 41.

### Electron-induced light emission mapping

Light emitted as a consequence of electron interaction with samples is collected by a parabolic mirror located above the sample, so as to be confocal with the incident electron beam (which passes through a small hole in the mirror), and directed to either a VIS/NIR spectrometer (Horiba iHR320 imaging spectrometer with nitrogen-cooled detector array) or a CCD camera configured to image the parabolic mirror surface, that is to map the angular distribution of light emission. Band-pass filters may be optionally inserted in the latter, imaging beam path. In this work we present as-recorded angle-resolved emission data for all samples, converted directly from the square CCD array to spherical coordinates without background subtraction or post-processing correction for instrumental asymmetry (the *x/y/z* position of the mirror is routinely adjusted to correctly centre and frame the CCD emission map image, but no tip/tilt adjustment is available for fine-tuning) – the need of which is negated by the brightness and directionality of holographic sample emission. The self-consistency of data sets (such as in Figs 3 and 4) against instrumental alignment artefacts is ensured through the use of fixed azimuthal and/or polar angles in each case.

### Data Availability

The data from this paper can be obtained from the University of Southampton ePrints research repository, DOI: 10.5258/SOTON/383663.

## Additional information

**How to cite this article**: Li, G. *et al*. Holographic free-electron light source. *Nat. Commun.* **7**, 13705 doi: 10.1038/ncomms13705 (2016).

**Publisher's note**: Springer Nature remains neutral with regard to jurisdictional claims in published maps and institutional affiliations.

## References

[b1] Kramper P. . Highly directional emission from photonic crystal waveguides of subwavelength width. Phys. Rev. Lett. 92, 113903 (2004). 15089137 10.1103/PhysRevLett.92.113903

[b2] Lezec H. J. . Beaming light from a subwavelength aperture. Science 297, 820–822 (2002). 12077423 10.1126/science.1071895

[b3] Greffet J.-J. . Coherent emission of light by thermal sources. Nature 416, 61–64 (2002). 11882890 10.1038/416061a

[b4] Yu N. . Small-divergence semiconductor lasers by plasmonic collimation. Nat. Photon. 2, 564–570 (2008).

[b5] Curto A. G. . Unidirectional emission of a quantum dot coupled to a nanoantenna. Science 329, 930–933 (2010). 20724630 10.1126/science.1191922

[b6] Lee K. G. . A planar dielectric antenna for directional single-photon emission and near-unity collection efficiency. Nat. Photon. 5, 166–169 (2011).

[b7] Novotny L. & van Hulst N. Antennas for light. Nat. Photon. 5, 83–90 (2011).

[b8] Bossard J. A. & Werner D. H. Metamaterials with angle selective emissivity in the near-infrared. Opt. Express 21, 5215–5225 (2013). 23482092 10.1364/OE.21.005215

[b9] Sreekanth K. V., Biaglow T. & Strangi G. Directional spontaneous emission enhancement in hyperbolic metamaterials. J. Appl. Phys. 114, 134306 (2013).

[b10] Denisyuk A. I. . Transmitting Hertzian optical nanoantenna with free-electron feed. Nano Lett. 10, 3250–3252 (2010). 20731411 10.1021/nl1002813

[b11] Coenen T., Vesseur E. J. R., Polman A. & Koenderink A. F. Directional emission from plasmonic yagi–uda antennas probed by angle-resolved cathodoluminescence spectroscopy. Nano Lett. 11, 3779–3784 (2011). 21780758 10.1021/nl201839g

[b12] Coenen T., Bernal Arango F., Femius Koenderink A. & Polman A. Directional emission from a single plasmonic scatterer. Nat. Commun. 5, 3250 (2014). 24488237 10.1038/ncomms4250

[b13] Adamo G. . Light well: a tunable free-electron light source on a chip. Phys. Rev. Lett. 103, 113901 (2009). 19792372 10.1103/PhysRevLett.103.113901

[b14] Adamo G. . Electron-beam-driven collective-mode metamaterial light source. Phys. Rev. Lett. 109, 217401 (2012). 23215613 10.1103/PhysRevLett.109.217401

[b15] Gabor D. A new microscopic principle. Nature 161, 777–778 (1948). 18860291 10.1038/161777a0

[b16] Bazhenov V. Y., Vasnetsov M. V. & Soskin M. S. Laser beams with screw dislocations in their wavefronts. JETP Lett. 52, 429–431 (1990).

[b17] Heckenberg N. R., McDuff R., Smith C. P., Rubinsztein-Dunlop H. & Wegener M. J. Laser beams with phase singularities. Opt. Quant. Electron. 24, S951–S962 (1992).

[b18] Verbeeck J., Tian H. & Schattschneider P. Production and application of electron vortex beams. Nature 467, 301–304 (2010). 20844532 10.1038/nature09366

[b19] Voloch-Bloch N., Lereah Y., Lilach Y., Gover A. & Arie A. Generation of electron airy beams. Nature 494, 331–335 (2013). 23426323 10.1038/nature11840

[b20] Ginzburg V. L. & Frank I. M. Radiation of a uniform moving electron due to its transition from one medium into another. J. Phys. (USSR) 9, 353–362 (1945).

[b21] García de Abajo F. J. Optical excitations in electron microscopy. Rev. Mod. Phys. 82, 209–275 (2010).

[b22] Brenny B. J. M., Coenen T. & Polman A. Quantifying coherent and incoherent cathodoluminescence in semiconductors and metals. J. Appl. Phys. 115, 244307 (2014).

[b23] Lukosz W. & Kunz R. E. Light emission by magnetic and electric dipoles close to a plane interface. I. Total radiated power. J. Opt. Soc. Am. 67, 1607–1615 (1977).

[b24] Hansen P. M. *The radiation efficiency of a dipole antenna located above an imperfectly conducting ground* Doctoral thesis, University of Michigan (1970).

[b25] Brown B. R. & Lohmann A. W. Computer-generated binary holograms. IBM J. Res. Dev. 13, 160–168 (1969).

[b26] Lee W.-H. Binary synthetic holograms. Appl. Opt. 13, 1677–1682 (1974). 20134530 10.1364/AO.13.001677

[b27] Krasavin A. V., MacDonald K. F. & Zheludev N. in *Nanophotonics with Surface Plasmons Advances in Nano-Optics and Nano-Photonics* (eds Shalaev, V.M. & Kawata, S.) 109–139 (Elsevier, 2007).

[b28] Bashevoy M. V., Jonsson F., MacDonald K. F., Chen Y. & Zheludev N. I. Hyperspectral imaging of plasmonic nanostructures with nanoscale resolution. Opt. Express 15, 11313–11320 (2007). 19547488 10.1364/oe.15.011313

[b29] Suzuki T. & Yamamoto N. Cathodoluminescent spectroscopic imaging of surface plasmon polaritons in a 1-dimensional plasmonic crystal. Opt. Express 17, 23664–23671 (2009). 20052076 10.1364/OE.17.023664

[b30] Osorio C. I., Coenen T., Brenny B. J. M., Polman A. & Koenderink A. F. Angle-Resolved Cathodoluminescence Imaging Polarimetry. ACS Photon. 3, 147–154 (2015).

[b31] Sapienza R. . Deep-subwavelength imaging of the modal dispersion of light. Nat. Mater. 11, 781–787 (2012). 22902895 10.1038/nmat3402

[b32] Merano M. . Probing carrier dynamics in nanostructures by picosecond cathodoluminescence. Nature 438, 479–482 (2005). 16306988 10.1038/nature04298

[b33] Tizei L. H. G. & Kociak M. Spatially resolved quantum nano-optics of single photons using an electron microscope. Phys. Rev. Lett. 110, 153604 (2013). 25167267 10.1103/PhysRevLett.110.153604

[b34] Bashevoy M. V. . Generation of traveling surface plasmon waves by free-electron impact. Nano Lett. 6, 1113–1115 (2006). 16771563 10.1021/nl060941v

[b35] Kuttge M. . Loss mechanisms of surface plasmon polaritons on gold probed by cathodoluminescence imaging spectroscopy. Appl. Phys. Lett. 93, 113110 (2008).

[b36] Nye J. F. & Berry M. V. Dislocations in wave trains. Proc. R. Soc. Lond. A 336, 165–190 (1974).

[b37] He H., Friese M. E. J., Heckenberg N. R. & Rubinsztein-Dunlop H. Direct observation of transfer of angular momentum to absorptive particles from a laser beam with a phase singularity. Phys. Rev. Lett. 75, 826–829 (1995). 10060128 10.1103/PhysRevLett.75.826

[b38] Grier D. G. A revolution in optical manipulation. Nature 424, 810–816 (2003). 12917694 10.1038/nature01935

[b39] Kotlyar V. V. . Generation of phase singularity through diffracting a plane or Gaussian beam by a spiral phase plate. J. Opt. Soc. Am. A 22, 849–861 (2005). 10.1364/josaa.22.000849 15898544

[b40] Yu N. . Light propagation with phase discontinuities: generalized laws of reflection and refraction. Science 334, 333–337 (2011). 21885733 10.1126/science.1210713

[b41] Johnson P. B. & Christy R. W. Optical constants of the noble metals. Phys. Rev. B 6, 4370–4379 (1972).

